# Investigation of the Longitudinal Relaxation Time of Rat Tibial Cortical Bone Using SWIFT

**DOI:** 10.2463/mrms.tn.2016-0050

**Published:** 2016-12-12

**Authors:** Tsuyoshi Sukenari, Kazuya Ikoma, Motoyuki Horii, Masahiro Umeda, Masamitsu Kido, Shigeki Hayashi, Yusuke Hara, Tetsuro Yamasaki, Okihiro Onishi, Toru Morihara, Hiroyoshi Fujiwara, Mitsuhiro Kawata, Toshikazu Kubo

**Affiliations:** 1 Department of Orthopaedics, Graduate School of Medical Science, Kyoto Prefectural University of Medicine, 465 Kajii-cho Kawaramachi-Hirokoji, Kamigyo-ku, Kyoto 602-8566, Japan; 2 Department of Anatomy and Neurobiology, Graduate School of Medical Science, Kyoto Prefectural University of Medicine, Kyoto, Japan; 3 Department of Medical Informatics, Meiji University of Integrative Medicine, Kyoto, Japan

**Keywords:** cortical bone, longitudinal relaxation time, sweep imaging with Fourier transform

## Abstract

Sweep imaging with Fourier transform (SWIFT) method has been developed to image tissues with very short T_2_ values, such as cortical bone. The purpose of this study was to measure the T_1_ value of the rat cortical bone. It was approximately 120 ms on 7.04T. This result could thus be useful for studying bony tissue according to the SWIFT method in the future.

## Introduction

Various imaging methods including X-ray, computed tomography (CT), magnetic resonance imaging (MRI) and radioisotope examinations are used to diagnose musculoskeletal disease. MRI is a particularly non-invasive diagnostic imaging method that detects the protons of tissues with high contrast resolution, making it a useful tool for evaluating musculoskeletal disease. X-rays and CT are also valuable imaging methods for assessing diseases involving changes in cortical bone, such as osteoporosis, in which conventional MRI is not helpful. This is because the protons of cortical bone have a very short transverse relaxation time (T_2_) value (< 1 ms),^1–3^ which causes the signals from the protons in cortical bone to rapidly decay, thus making it difficult to detect these signals and image cortical bone on conventional MRI sequences.

In recent years, sweep imaging with Fourier transform (SWIFT) method and ultra-short echo time (UTE) imaging method have been developed to image tissues with very short T_2_ values, such as cortical bone.^4–8^ Using the SWIFT method, the echo time (TE) can theoretically be reduced to nearly zero; therefore, almost all proton signals in the cortical bone may be detected, thus making such imaging possible. It is important to investigate the longitudinal relaxation time (T_1_) of normal cortical bone to determine the MRI parameters for the cortical bone and to make it possible to diagnose various bone diseases clinically. Indeed, the variable flip angle SWIFT (VFA-SWIFT) method has been used to accurately determine the T_1_ of iron oxide nanoparticle suspensions^9^ and osteochondral specimens.^10^ For these reasons, it is hoped that the VFA-SWIFT method can allow for accurate measurement of the T_1_ of the cortical bone.

The purpose of this study was to measure the T_1_ value of the cortical bone in rat tibias using the VFA-SWIFT method.

## Materials and Methods

### Measurement of the phantom T_1_ values

The uniformity of the B_1_ field by 0.1 mM manganese chloride (MnCl_2_) was measured using the fast spin echo method with a transmit/receive surface coil (4 × 3 cm in diameter) and a high magnetic field MRI unit used for animal experiments (Varian 7.04 Tesla [T] MRI system; Agilent Technologies Inc., Palo Alto, California, USA). A columnar container measuring 1.5 cm in diameter including 0.1 mM MnCl_2_ was placed in the center of the surface coil under a 22°C room temperature. The uniformity of the B_1_ field was tested in a preliminary experiment using 0.1 mM MnCl_2_ and the uniformity was 95.4% calculated by the National Electrical Manufacturers Association (NEMA) equation (Eq.).^11^ Thus, the B_1_ field variation in the container was confirmed to be negligible.

The T_1_ values of 6.0 mM MnCl_2_ were measured according to the VFA method using the gradient echo (GRE) and the SWIFT method with the same surface coil and the same MRI unit. A columnar container measuring 0.5 cm in diameter including 6.0 mM MnCl_2_ was placed in the center of the surface coil under a room temperature of 22°C. The GRE three-dimensional method was subsequently carried out under conditions of a changing flip angle (FA) with 5 to 90°(changing every 5°) using the following parameters: repetition time (TR), 12.5 ms; TE, 1.39 ms; average, 4; dummy scans, 0; matrix, 128 × 128 × 128; field of view (FOV), 80 × 80 × 80 mm^3^; resolution, 0.625 × 0.625 × 0.625 mm^3^; bandwidth, 100 kHz; radio frequency (RF) pulse width, 1,000 μs; acquisition time, 1.28 ms; and imaging time, 13 min 39 sec. The region of interest (ROI) in the shape of a circle was set to include 75% of the whole phantom area around the center of the phantom.^12^ The mean signal intensity (SI) in the ROI was measured.^6^

The SI on spoiled GRE imaging and SWIFT is determined from the T_1_ and T_2_ values of the proton signal, TR, TE and FA and represented by the following Eq. 1;^8^

(1) $$\begin{array}{l} \text{SI}\propto \text{exp}\left(-\text{TE}/{\text{T}}_{2}^{*}\right)\left\{1-\text{exp}\left(-\text{TR}/{\text{T}}_{1}\right)\right\}\\ \;\;\;\;\;\left[\text{sin}(\text{FA})/\left\{1-\text{cos}(\text{FA})\text{exp}\left(-\text{TR}/{\text{T}}_{1}\right)\right\}\right] \end{array}$$

The SWIFT method was carried out under conditions of changing FA^4,13^ with 5 to 90° (changing every 5°) using the following parameters: TR, 12.5 ms; spirals, 16; matrix, 256 × 256 × 256; views, 8,192; average, 1; dummy scans, 512; FOV, 80 × 80 × 80 mm^3^; resolution, 0.313 × 0.313 × 0.313 mm^3^; bandwidth, 62.5 kHz; acquisition time, 4.096 ms; imaging time, 27 min 25 sec; and pulse type, hyperbolic secant pulse. The ROI was set in the same manner as that described for the GRE method. The mean SI in the ROI was measured.

The SI on spoiled GRE imaging is expressed by Eq. 2 referred to Eq. 1 if TE is nearly equal to zero.

The SI determined according to the SWIFT method is expressed by Eq. 2 referred to Eq. 1 because SWIFT is not influenced by transverse relaxation:^4^

(2) $$\begin{array}{l} \text{SI}={\text{M}}_{0}\times \text{sin}(\text{FA})\times \left\{1-\text{exp}\left(-\text{TR}/{\text{T}}_{1}\right)\right\}/\\ \;\;\;\;\;\;\;\;\;\;\;\left\{1-\text{exp}\left(-\text{TR}/{\text{T}}_{1}\right)\times \text{cos}(\text{FA})\right\} \end{array}$$

where M_0_ is spin density.

In the SWIFT method, the FA for the maximal SI under the conditions of a fixed TR was regarded as α_E_ and depends on the T_1_ value. It is expressed by Eq. 3:^4^

(3) $$\text{cos}\alpha_{\text{E}}=\text{exp}\left(-\text{TR}/{\text{T}}_{1}\right)$$

The SI obtained from 6.0 mM MnCl_2_ was linearized according to Eq. 2 and 3. The horizontal axis was SI × cos (FA)/sin (FA) and the vertical axis was SI/sin (FA) expressed by Eq. 4.

(4) $$\begin{array}{ll} \text{SI}/\text{sin}(\text{FA}) & =\text{exp}\left(-\text{TR}/{\text{T}}_{1}\right)\times \left\{\text{SI}\times \text{cos}(\text{FA})/\text{sin}(\text{FA})\right\}\\ & +{\text{M}}_{0}\times \left\{1-\text{exp}\left(-\text{TR}/{\text{T}}_{1}\right)\right\} \end{array}$$

Curve fitting was performed using the fittype (ax+b) function of the MATLAB software program (MathWorks; Natick, Massachusetts, USA). The T_1_ value of 6.0 mM MnCl_2_ in each voxel was calculated from the fitted slope, exp (−TR/T_1_), and the T_1_ map was made. The T_1_ values were obtained from Eq. 4.

6.0 mM MnCl_2_ was collected at 7T using an AVANCE III NMR spectrometer (Bruker Biospin, Baden-Württemberg, Germany) with a 10-mm bird-cage RF coil. A one ml solution of MnCl_2_ was placed in a 10-mm NMR tube. The temperature was maintained at 22°C. The T_1_ value of the MnCl_2_ solution was measured by the inversion-recovery (IR) pulse sequence (RD - 180° - VD - 90° - acquisition of FID), where RD is the relaxation delay of 3 sec, 180° RF pulse is 69 μs of block-pulse, VD is variable delay, 90° RF pulse is 34.5 μs of block-pulse, and FID is free induction decay. VD was changed from 1 ms to 300 ms, and the T_1_ value was measured. In addition, VD was set at 3000 ms and then Mo was measured. The T_1_ values were calculated based on the SI at VD (Mv) using Eq. 5, as follows:

(5) $$\text{log}\left\{\left(\text{Mo}-\text{Mv}\right)/\left(2\text{Mo}\right)\right\}=1/{\text{T}}_{1}\times \text{VD}$$

where Mo is the SI obtained with a VD of 3000 ms.

### Measurement of the T_1_ value of the cortical bone in the rat tibias

This study evaluated five lower thighs obtained from five 12-week-old female Sprague-Dawley wild-type rats (Shimizu Laboratory Supplies; Kyoto, Japan) raised in an animal house at our institution in accordance with the policies and procedures set out in the “*Guidelines for the Care and Use of Laboratory Animals*” issued by the National Institutes of Health. This study was approved by the ethics review board for animal experiments at our institution.

The right tibias of the rats were extracted as a single lump together with the surrounding tissues and used as specimens. Each specimen was placed in a columnar container measuring 1.5 cm in diameter to take phantom images. The specimens were then immersed in a fluorine-based inert liquid (Fluorinert FC-3283^®^, Sumitomo 3M, Tokyo Japan) to attenuate the artifacts with susceptibility. Imaging was subsequently carried out using the MRI unit and the surface coil as described for obtaining the phantom images. The container with the specimen inside was set in the center of the surface coil to avoid RF non-uniformity. The specimen was installed in the same direction to maintain the FA. Regarding the imaging conditions of the SWIFT method, the parameters were fixed at TR = 12.5 ms under conditions of a changing FA (*n* = 5) with 5 to 90° (changing every 5°). These settings were provided under the common imaging parameters used to obtain phantom images except for FOV and resolution. FOV was set at 40 × 40 × 40 mm^3^ and resolution was 0.156 × 0.156 × 0.156 mm^3^.

**Fig 1.**
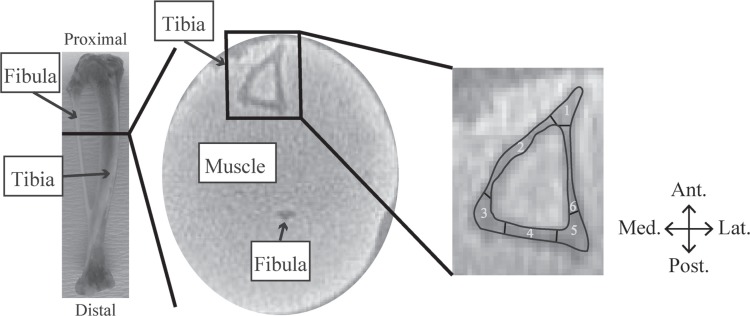
The set ROI surrounded by white line. Six ROIs, including all areas of the cortical bone, were set in the cortical bone in the diaphysis of the tibia with the transected image by SWIFT method. ROI: region of interest, SWIFT: sweep imaging with Fourier transform.

Upon imaging, six ROIs, including all areas of the cortical bone, were set in the cortical bone in the diaphysis of the tibia with the same transected image (Fig. 1). Subsequently, the mean SI in the ROIs was measured, and the mean value was obtained. Curve fitting was performed using the MATLAB software program, and the T_1_ map of the cortical bone was made as well as 6.0 mM MnCl_2_. The T_1_ values were obtained from Eq. 4.

The mean SI and the mean T_1_ value of five cortical bones were expressed as the mean ± standard deviation.

## Results

### Measurement of the phantom T_1_ values

**Fig 2.**
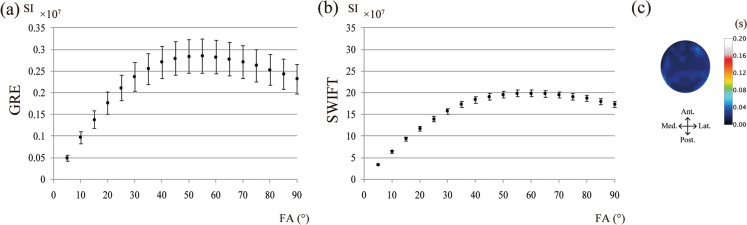
Changes in the SI of the 6.0 mM MnCl_2_ based on the changes in the FA based on the GRE (**a**) and SWIFT (**b**) methods and the T_1_ map obtained using the SWIFT method (**c**). The FA for the SI of 6.0 mM MnCl_2_ reached a maximum at 55° using GRE (**a**) and 60° using SWIFT (**b**). The T_1_ value on the map obtained according to the SWIFT method closely corresponded to the T_1_ value obtained from Eq. 4 (**c**). SI: signal intensity, FA: flip angle, GRE: gradient echo, SWIFT: sweep imaging with Fourier transform.

When using the GRE method, the FA for the SI of 6.0 mM MnCl_2_ reached a maximum at 55° (Fig. 2a). The T_1_ value was 23.9 ms obtained from Eq. 4.

When using the SWIFT method, the FA for the SI of 6.0 mM MnCl_2_ reached a maximum at 60° (Fig. 2b). The T_1_ value was 20.8 ms obtained from Eq. 4. The T_1_ value obtained using the SWIFT method was slightly smaller than those obtained with the GRE method.

The T_1_ value was 26.1 ms obtained by means of the spectroscopic IR method.

The T_1_ value on the map closely corresponded to the T_1_ value obtained from Eq. 4 according to the SWIFT method (Fig. 2c).

### Measurement of the T_1_ value of the cortical bone in the rat tibias

**Fig 3.**
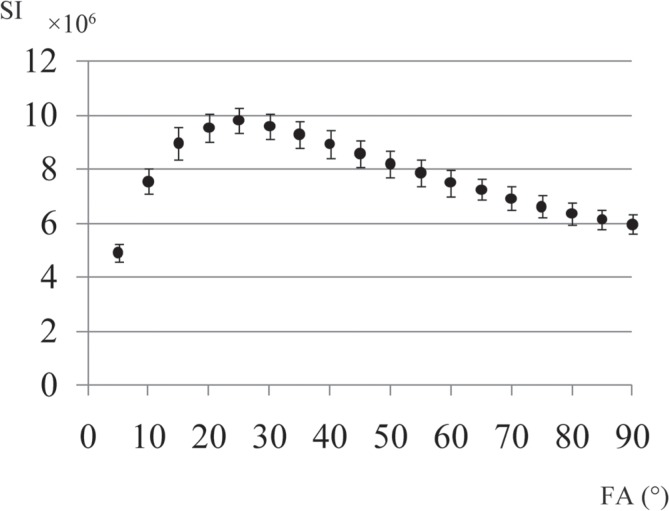
Changes in the SI of the tibial cortical bone based on the changes in the FA. The cortical bone SI reached a maximum when the FA was 25° in all cases. The mean cortical bone SI was (9.8 ± 0.45) × 10^6^ at FA = 25° and TR = 12.5 ms. SI: signal intensity, FA: flip angle, TR: repetition time.

**Fig 4.**
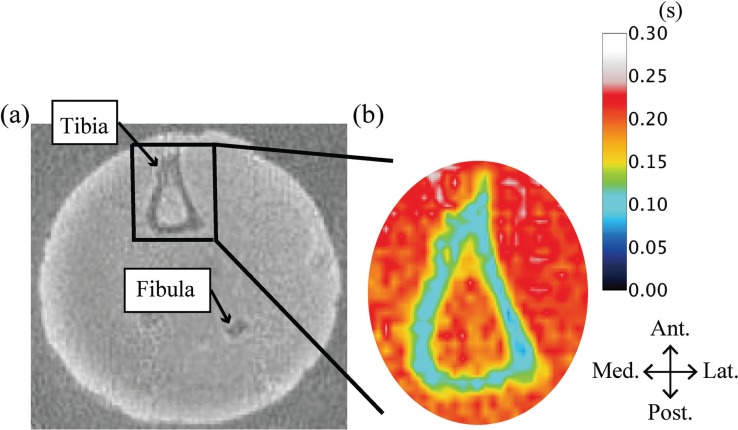
SWIFT transected image (**a**) and the T_1_ map (**b**) of the lower thigh tissue of a rat. Signals from the bony tissue are shown, and a clear contrast was obtained. The T_1_ value on the map closely corresponded to the T_1_ value obtained from Eq. 4. SWIFT: sweep imaging with Fourier transform.

Under the conditions of a changing FA, the cortical bone SI reached a maximum with an FA of 25° in all cases (Fig. 3). The mean cortical bone SI was (9.8 ± 0.45) × 10^6^ at FA = 25° and TR = 12.5 ms. As shown in Fig. 4 for a case, the signals from the bony tissues were imaged and a clear contrast was obtained. The mean T_1_ value of five cortical bones was 117.6 ± 7.25 ms obtained from Eq. 4. The T_1_ value on the map closely corresponded to the T_1_ value obtained from Eq. 4.

## Discussion

It is expected that signals from connective tissues may be non-invasively rendered using the SWIFT method. Obtaining an accurate measurement of the cortical bone SI is necessary for performing accurate cortical bone T_1_ measurements, and it is important to clearly acquire cortical bone images. In this study, rats commonly used in basic research on bone disease were assessed. To the best of our knowledge, there are no past reports of the cortical bone T_1_ values in rats. It is difficult to obtain the robust SI for the cortical bone of rats *in vivo* because the animals are very small. The water content of cortical bone, muscle and fat in humans is approximately 15%, 70% and 90%, respectively,^6,7^ with the water content of cortical bone being overwhelmingly less than that of muscle and fat. The T_1_ value of cortical bone depends on bone water status.^14^ Therefore, fresh tibias surrounded by muscle and fat as one lump were imaged *ex vivo* to prevent dryness of the bony tissues and obtain a good cortical bone SI in addition to better tissue contrast. Future studies are needed to identify the best conditions for achieving a higher contrast of cortical bone in small specimens *in vivo* using the SWIFT method.

The T_1_ value can be measured using the IR method, the saturation recovery method or the VFA method. The imaging time is longer for the IR method and the saturation recovery method but shorter for the VFA method, as it only measures the SI at two FAs across the Ernst angle. However, the SI was measured for multiple FAs and using a long TR to obtain a more accurate T_1_ value for MnCl_2_ and cortical bone in this study, because the first and basic study assessed the cortical bone T_1_ value using the VFA-SWIFT method. Therefore, the scan time in this study was too long for *in vivo* and clinical applications. Reducing the number of FAs and/or shortening the TR is a simple and straightforward way of reducing the scan time. It was noted that the cortical bone SI reached its maximum with an FA of 25° in all cases in this study. The cortical bone SI should therefore be measured at FAs across 25°, such as from 15° to 36° (changing every 3°), to measure the T_1_ value of cortical bone. Moreover, future studies must ensure that a better SI is obtained for a shorter TR with the VFA-SWIFT method.

The higher the magnetic field, the longer the T_1_ value of the tissues becomes. However, the T_1_ value of cortical bone on 7.04T could not be found based on extensive reading of previous reports and the calculated values could not be compared with the results of this study. The T_1_ value of both 6.0 mM MnCl_2_ obtained from the SWIFT method closely corresponded to that obtained from the GRE and spectroscopic IR method. However, the T_1_ value of 6.0 mM MnCl_2_ was slightly shorter according to the SWIFT method than that obtained using the GRE and the spectroscopic IR method. This could be because more signals, especially low signals due to short T_2_, are obtained from the phantom using the SWIFT method versus the GRE and spectroscopic IR method.^10^ The GRE and spectroscopic IR method are affected by transverse relaxation. In the SWIFT method, the TE is set to nearly zero, and swept radiofrequency excitation and signal acquisition are performed at almost the same time; therefore, SWIFT minimizes the effects from signal decay due to transverse relaxation, and the T_1_ values of various tissues having short T_2_ values, including cortical bone, can be measured easily and exactly. Springer et al. reported that the T_1_ value of the human tibial cortical bone is approximately 80 ms using the VFA method on 3T MRI.^8^ On the other hand, Du et al. reported that the T_1_ value of the human tibial cortical bone is approximately 230 ms using the saturation recovery method on 3T MRI.^6^ The T_1_ value of the rat tibial cortical bone was found to be 118 ms according to the VFA method in this study. This result is partially consistent with the findings of past reports considering that a higher magnetic field was used and there was a longer T_1_ value for the tissues although the species were different. The cortical bone water status, upon which the T_1_ value depends,^14^ may differ based on the condition, and measuring the T_1_ value of cortical bone remains challenging.

This study is associated with various limitations. First, the measurement accuracy was limited due to the small size of the specimens, the low SI of cortical bone, affected the T_1_ value accuracy. Future studies are needed to investigate the cortical bones of large animals. Second, bullseye artifacts are generated in the center in order to radially supplement the k-space^13^ and the SI of this site cannot be measured. However, bullseye artifacts can be avoided by having the measured site be slightly off center, thereby achieving a good ROI setting. Third, a surface coil with considerable B_1_ field variation in space and *ex vivo* specimens were used due to the MRI system conditions. T_1_ measurements obtained using the VFA-SWIFT method are sensitive to B_1_ field inhomogeneity and FA error.^15^ However, with regard to the SWIFT method, there have been only a few reports in which images of an *ex vivo* specimen were examined primarily, and such studies are still in the preclinical stage.^4,13,16^ If optimal imaging conditions for assessing cortical bone with the SWIFT method using volume coil and the Look-Locker method can be determined, such results may facilitate *in vivo* studies and obtain a more accurate T_1_ value of cortical bone.^15^ We believe that our present data help to support the use of this method and provide evidence that obtaining better resolution is also possible.

The SWIFT method gradually changes the magnetic field gradient pulse; therefore, this method is advantageous in that the sound during the examination is quiet and the TE is substantially zero with little motion or flow artifacts. Although SWIFT is associated with some problems such as a high RF power and a specific absorption rate limitation for *in vivo* studies, the clinical application of this modality is nevertheless anticipated.

## Conclusion

We herein investigated the T_1_ value of the rat tibial cortical bone using the SWIFT method. The T_1_ value of the rat tibial cortical bone was approximately 120 ms on 7.04T MRI. This result could be useful for studying bony tissue using the SWIFT method in the future.
